# The Role of Cannabinoids as Anticancer Agents in Pediatric Oncology

**DOI:** 10.3390/cancers13010157

**Published:** 2021-01-05

**Authors:** Clara Andradas, Alexandra Truong, Jacob Byrne, Raelene Endersby

**Affiliations:** 1 Brain Tumour Research Program, Telethon Kids Institute, Nedlands, WA 6009, Australia; 22725235@student.uwa.edu.au (A.T.); Jacob.Byrne@telethonkids.org.au (J.B.); 2 Centre for Child Health Research, University of Western Australia, Nedlands, WA 6009, Australia

**Keywords:** cannabinoid, medical cannabis, Δ9-tetrahydrocannabinol, THC, CBD, cannabidiol, pediatric oncology, childhood cancer

## Abstract

**Simple Summary:**

The endocannabinoid system (ECS) is a complex signaling pathway system involved in the regulation of multiple functions in both normal tissues and cancer. Δ9-tetrahydrocannabinol and cannabidiol are plant-derived cannabinoids that possess some efficacy against adult cancer, in part via modulation of the ECS, and may be less toxic agents compared to other treatments used in oncology. To date, there are minimal studies that have investigated these drugs in the pediatric cancer setting. Indeed, there are currently no preclinical or clinical studies examining the effects of cannabinoids in pediatric brain cancer, although there is some evidence that they can alleviate symptoms associated with childhood cancer therapy, such as vomiting and nausea. Given there is accumulating evidence that cannabis use during adolescence is associated with poor mental and cognitive health, there is a present and urgent need to investigate the safety and efficacy of cannabinoids in pediatric oncology to provide guidance to families and physicians.

**Abstract:**

Cannabinoids are a group of chemicals that bind to receptors in the human body and, in turn, modulate the endocannabinoid system (ECS). They can be endogenously produced, synthetic, or derived from the plant *Cannabis sativa L*. Research over the past several decades has shown that the ECS is a cellular communication network essential to maintain multiple biological functions and the homeostasis of the body. Indeed, cannabinoids have been shown to influence a wide variety of biological effects, including memory, pain, reproduction, bone remodeling or immunity, to name a few. Unsurprisingly, given these broad physiological effects, alterations of the ECS have been found in different diseases, including cancer. In recent years, the medical use of cannabis has been approved in different countries for a variety of human conditions. However, the use of these compounds, specifically as anticancer agents, remains controversial. Studies have shown that cannabinoids do have anticancer activity in different tumor types such as breast cancer, melanoma, lymphoma and adult brain cancer. Specifically, phytocannabinoids Δ9-tetrahydrocannabinol (THC) and cannabidiol (CBD) has been shown to induce apoptosis and inhibit proliferation of adult cancer cells, as well as modulate angiogenesis and metastasis. Despite increasing evidence that cannabinoids elicit antitumor effects in adult cancers, there is minimal data available on their effects in children or in pediatric cancers despite public and clinical demand for information. Here we describe a comprehensive and critical review of what is known about the effects of cannabinoids on pediatric cancers, highlight current gaps in knowledge and identify the critical issues that need addressing before considering these promising but controversial drugs for use in pediatric oncology.

## 1. Introduction

Throughout history, cannabinoids have featured ubiquitously in modern and ancient cultures for their potent biological and psychoactive properties, giving rise to their therapeutic use in folk medicine. Although more than 100 cannabinoids derived from the plant *Cannabis sativa* (phytocannabinoids) have been identified [1], Δ9-tetrahydrocannabinol (THC) and cannabidiol (CBD) attract the most attention due to their natural abundance and potency. These compounds act via the endocannabinoid system (ECS), which was originally discovered through research to understand the psychoactive effects of THC. This system has broad-reaching effects in mammals and is comprised of two G-protein coupled cannabinoid receptors type 1 and 2 (CB_1_R and CB_2_R), their endogenous cannabinoid ligands (endocannabinoids) and the enzymes which regulate their synthesis and degradation [2].

**Figure 1:**
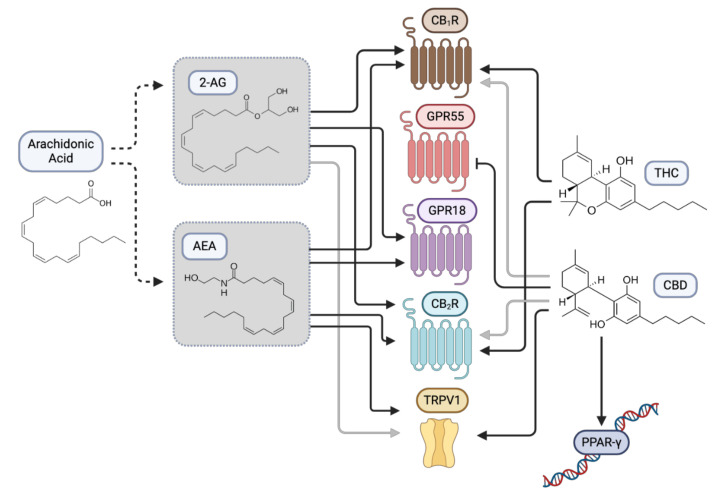
Plant-derived cannabinoids modulate cannabinoid and non-cannabinoid receptors in the human body. Δ9-tetrahydrocannabinol (THC) functions primarily by activating G-protein coupled cannabinoid receptors type 1 and 2 (CB_1_R and CB_2_R) that are normally stimulated by endogenous cannabinoids, such as N-arachidonoylethanolamine (AEA) and 2-arachidonoylglycerol (2-AG). Additionally, phytocannabinoids have been shown to bind and modulate the activities of non-cannabinoid receptors. Examples shown are G-protein coupled receptors including GPR55 and GPR18, transient receptor potential channels (e.g., TRPV1) and peroxisome proliferator-activated receptors (e.g., PPARγ).

The best-characterized endocannabinoids are 2-arachidonoylglycerol (2-AG) and N-arachidonoylethanolamide (AEA or anandamide). They are lipid-based signaling molecules that are synthesized from arachidonic acid present in the cellular membrane [2]. These endocannabinoids mediate different biological functions by binding and stimulating CB_1_R and CB_2_R [3,4]. Both receptors are expressed throughout the body with abundant expression of CB_1_R in the central nervous system (CNS) [5] and CB_2_R primarily found in immune cells with some cell-specific CNS expression [6]. While many effects of THC are mediated via CB_1_R and CB_2_R, CBD has a lower affinity for these receptors. Additionally, endocannabinoids and phytocannabinoids can bind and mediate their effects by modulating non-cannabinoid receptors such as adenosine receptor, transient receptor potential cation channel subfamily V member 1 (TRPV1), peroxisome proliferator-activated receptors (PPARs) and other G-protein coupled receptors including GPR55 and GPR18 [7,8,9] (Figure 1).

The characterization of CB_1_R in the early 1990s led to the discovery that the ECS is involved in several pathophysiological processes, including embryogenesis, neurogenesis, immune response, memory and learning, metabolism, and inflammation, to name a few [10]. In consequence, immense interest in whether the ECS could be therapeutically targeted for the human disease developed. In this context, one promising avenue of research has been the discovery of THC and CBD as anticancer agents. Over the past two decades, preclinical data indicate that THC, CBD, as well as other synthetic cannabinoids induce cancer cell death and inhibit tumor proliferation, metastasis, and angiogenesis [11]. While it is generally accepted that cannabinoid-based treatments confer relatively few negative side effects in adult cancer patients compared to conventional treatments such as radiotherapy and chemotherapy, these results have unfortunately not been translated into many adult clinical trials despite increasing interest in these compounds in the medical and oncology space.

In terms of pediatric cancer, there is a paucity of clinical and preclinical evidence describing the pros and cons of medicinal cannabinoids. Indeed, recent surveys reveal only 30% of oncologists and pediatricians felt they had sufficient knowledge to make a qualified decision on the administration of cannabinoids to a sick patient [12], and 85% stated they needed more education on the safety and efficacy of cannabinoid treatments [13]. Despite this lack of self-reported expertise, 76% of 1446 oncologists said they would approve cannabinoids for a medical purpose [14,15]. Furthermore, despite the lack of rigorous research on cannabinoids in pediatric oncology, many parents still choose to administer cannabis to their sick children [16].

It is clear that there is a dearth of knowledge regarding the safety and efficacy of cannabinoids in pediatric oncology, and few guidelines exist that define or recommend effective dosing [17]. Here, we discuss what is known about the effects of cannabinoids on pediatric cancers and highlight current gaps in our knowledge. Specifically, we will focus on how the ECS is altered in pediatric cancers and which data are available about the antitumor effect of cannabinoids in these cancers, paying special attention to pediatric brain tumors given the promising data emerging on their utility in adult brain cancer. We aim to provide a better picture of the current knowledge regarding the potential use of cannabinoids for the treatment of pediatric cancers, the unsolved problems, and what future evidence is needed before considering these promising but controversial drugs for use in pediatric oncology.

## 2. Effects of Cannabinoids in Pediatric Cancer

**Figure 2:**
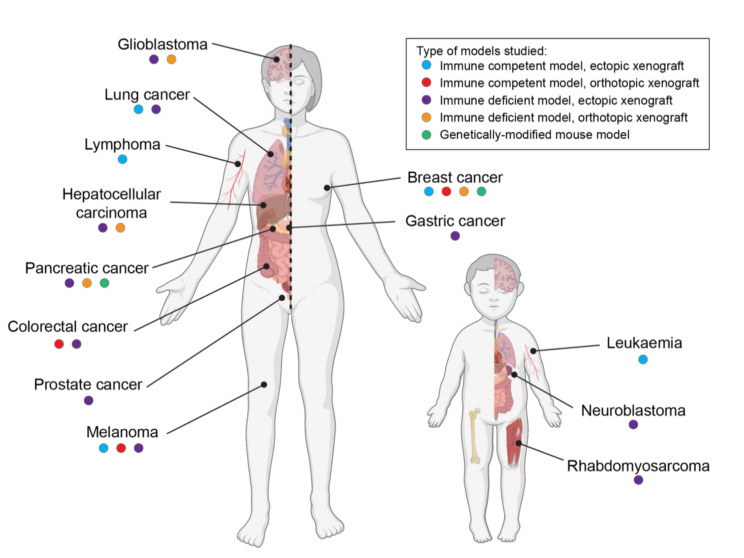
The anticancer activity of phytocannabinoids THC and cannabidiol (CBD) has been extensively demonstrated in animal models of adult cancer, but childhood cancers remain under-investigated. A range of animal studies including ectopic (blue dot) and orthotopic xenografts (including chemically induced tumors, red dot) in immune-competent mice; ectopic (purple dot) and orthotopic (orange dot) xenografts in immune-deficient mice; or studies on tumors spontaneously arising in genetically engineered mouse models (green dot) have been used to demonstrate the antitumor effects of THC and CBD. In contrast, preclinical research for pediatric cancers is limited to a few studies in a limited number of cancers. Of note, some animal studies have demonstrated that THC may also have tumor-promoting actions in lung and breast cancers.

In past decades, a broad range of studies has shown that cannabinoids exhibit antitumor properties in different adult cancer types, including breast cancer, melanoma, pancreatic cancer, lymphoma and brain tumors, among others. These include studies in a wide variety of experimental models of cancer, ranging from cancer cell lines to xenografted animals and genetically engineered mouse models [11,18] (Figure 2 and Table S1). Despite these data demonstrating the antitumor properties of cannabinoids in adult cancers, very little is known about their effects on pediatric tumors.

The majority of the research regarding pediatric cancers has been conducted in leukemia models, especially in T-cell acute lymphoblastic leukemia (T-ALL), a highly aggressive and chemotherapy-resistant cancer which makes up 15% of all childhood ALL cases. Several groups have shown that cannabinoids induce leukemia cell death both in vitro and in vivo [19,20,21,22,23,24]. Specifically, these studies show that cannabinoids increase intracellular stress and damage mitochondrial membrane potential, resulting in subsequent cytochrome c release and cleavage of caspases 8, 9, 2 and 10 [19,23,24]. Importantly, ceramide biosynthesis was shown to be essential for cannabinoid-mediated activation of the intrinsic apoptotic pathway [22], as also has been reported in adult glioblastoma models [25]. Additionally, CBD induces ROS production in leukemia cells [26], a common mechanism of action found in other cancers involving an increase in the expression of NAD(P)H oxidases Nox4 and p22^phox^ [24].

More recently, Kalenderoglou et al. showed that CBD targets the mammalian target of rapamycin (mTOR) pathway in leukemia cells, decreasing the phosphorylation of AKT, mTOR and ribosomal S6, affecting leukemia cell size [27]. In addition, Olivas-Aguirre and colleagues showed that CBD induces cell death via necrosis and autophagy in multiple types of T-ALL [20]. Suppose there is a direct link between the mTOR pathway, autophagy and apoptosis following cannabinoid treatment in leukemia cells, as there is in glioblastoma [25,28] or hepatocellular carcinoma [29]; it is still to be investigated. Importantly, promising results have been found when combining cannabinoids with leukemia chemotherapies. Specifically, THC and CBD synergize with doxorubicin, vincristine and cytarabine in leukemia cells in vitro [30,31]. Validating these results in vivo would be an essential step towards the clinical translation of these exciting data.

Encouraging data were reported in pediatric rhabdomyosarcoma, the most common soft tissue sarcoma in children, in 2009, when THC and the synthetic cannabinoid HU-210 were observed to inhibit the growth of these tumors [32]. In vitro and in vivo results showed that THC and HU-210 induced CB_1_R-mediated apoptosis via the inhibition of AKT signaling and an increase in stress-associated transcription factor p8. This finding was consistent with the actions observed for cannabinoid-induced apoptosis in adult cancer cells [33].

This was followed by another group that investigated the synthetic cannabinoid, WIN 55,212-2, a potent CB_1_R agonist, as an antitumoral agent using a model of pediatric osteosarcoma [34]. In cultured osteosarcoma cell lines, WIN 55,212-2 induced cell cycle arrest and upregulated several hallmarks of endoplasmic reticulum stress such as GRP78, CHOP and TRB3, along with subsequent autophagy [34]. As above, these mechanisms of action for cannabinoid signaling are consistent with reports from adult cancers [25,28].

Fisher and colleagues investigated the effects of THC and CBD in pediatric neuroblastoma [35]. They reported that both THC and CBD significantly reduced neuroblastoma cell viability in vitro, and CBD impeded xenograft growth in vivo [35]. While the study did not elucidate a mechanism for the antitumoral effects of CBD, it was noted that CBD induced neuroblastoma cell apoptosis both in vitro and in vivo.

Overall, these preclinical data indicate cannabinoids have potential anticancer efficacy across a range of different pediatric cancers, albeit with a variety of mechanisms of action reported. Of note, these pediatric cancers have very different cells of origin, exist within different tissue contexts and are typically driven by tumor-specific driver mutations [36]. The disadvantage of these studies is the use of long-term cultured cell lines, which are often criticized for not truly representing human cancer [37]. Few studies have validated their data in animal models, and those that did failed to use orthotopically xenografted models, thus, not replicating the true tissue context of these cancers (Figure 2 and Supplementary Table S1). Nonetheless, across these studies and despite the investigation of several different CB_1_R and/or CB_2_R agonists, cannabinoids appear to consistently reduce pediatric cancer cell proliferation. There are still numerous pediatric cancers where the effects of cannabinoids have not been examined, including brain cancer. Given the evidence demonstrating the antitumor effect of cannabinoids in adult brain tumors, the suitability of these compounds for childhood brain cancer treatment should be studied.

## 3. Cannabinoids and Pediatric Brain Tumors

Brain cancers comprise the second most common neoplasms diagnosed in children [38]. Numerous studies have confirmed an antitumor effect of cannabinoids in adult brain tumors. In mouse models of glioblastoma (the most common brain cancer in adults), it has been shown that both THC and CBD improved animal survival when administered in combination with temozolomide, the standard-of-care chemotherapy [39,40]. Furthermore, when glioma cells were pretreated with THC or CBD, either in vitro or in vivo, they were sensitized to radiation-induced death resulting in prolonged survival of mice [41]. However, despite these encouraging data, and the known ability of cannabinoids to penetrate the blood-brain barrier, there is no existing preclinical data on the effect of these agents in pediatric brain tumor models.

Although there is published evidence that ECS expression is altered in adult brain tumors, whether it is altered in pediatric brain tumors compared to healthy brain tissue and whether this alteration influences tumor progression is limited to two previous studies, highlighting the urgent need to expand the current literature. An initial study investigated CB_2_R immunoreactivity across 25 pediatric brain tumors [42]. CB_2_R immunoreactivity was reported to be very high in pediatric astrocytic tumors such as glioblastoma (*n* = 3) and subependymal giant cell astrocytoma (*n* = 1), but low in embryonal brain tumors such as medulloblastoma (*n* = 2) and supratentorial primitive neuroectodermal tumor (PNET, *n* = 1). These data suggested that CB_2_R expression may correlate with the histopathological origin of the disease [42].

For CB_1_R expression, Sredni and colleagues investigated gene expression in 33 pediatric low-grade gliomas. Within their cohort, they compared sub-totally resected cases where tumors were either stable or had spontaneously involuted with cases that relapsed. They reported that CB_1_R expression was the best predictor of spontaneous involution (*p* = 0.007) [43]. Moreover, they confirmed that the expression of CB_1_R in the fetal brain and these childhood brain cancers are significantly higher than in the adult brain [43], as are many pediatric brain cancer-associated genes [44,45].

There is a lack of published reports describing the expression of other components of the ECS in pediatric cancer. Searches for studies investigating AEA, 2-AG, GPR55, GPR18, PPAR or TRPV1 in pediatric brain tumors revealed a complete lack of research in this area. In contrast, for adult brain cancers, AEA levels have been studied in glioma, but with conflicting results. Some reported AEA to be higher in meningioma and glioma compared to normal brain tissue [46], while others found expression to be lower in glioma [47,48]. Conflicting results have also been reported for CB_1_R expression, with some authors demonstrating CB_1_R to be upregulated in high-grade glioma compared to low-grade glioma and normal brain tissue [47], while others have found the opposite [49]. Overall, a basic understanding of the role of the ECS system in pediatric brain cancer cells is required before one can speculate on the potential effects that cannabinoids may exert on these cancers, prior to considering their use in childhood CNS neoplasms.

## 4. Clinical Evidence of the Effect of Cannabinoids in Pediatric Brain Cancer

In line with the scarcity of preclinical data about the effect of cannabinoids in different pediatric cancer, to date, there are no clinical trials addressing the potential antitumor effect of cannabinoids in childhood cancer. However, there are anecdotal reports that tout the benefits of medicinal cannabis for children with brain cancer. A report by Foroughi et al. described two cases of spontaneous regression of low-grade glioma (pilocytic astrocytoma) in two females that coincided with the consumption of cannabis via inhalation [50]. As mentioned above, a retrospective study of low-grade gliomas shown that CB_1_R expression does correlate with tumor involution providing a plausible mechanism of action [43]; however, Foroughi and colleagues did not investigate CB_1_R expression in the cases reported.

In a more traditional approach, Kenyon and colleagues investigated the effects of synthetic CBD, delivered in controlled and defined doses to 119 patients with a broad range of different cancer types, including pediatric patients [51]. The best response to CBD was observed in a 5-year old male with posterior fossa ependymoma. After experiencing limited success with standard therapies, including two surgeries, chemotherapy and conformal photon radiotherapy, the child was prescribed CBD with no concomitant treatment. A magnetic resonance imaging scan performed 10 months after initiating CBD therapy revealed an approximately 60% decrease in tumor volume, and further scans showed stable disease [51]. Disease reduction was also reported in an adult with ependymoma, as well as for adults with breast, prostate and esophageal cancer. In this study, a maximal recommended clinical trial dose for CBD could not be defined due to the complete absence of side effects [51].

Despite numerous other anecdotal reports that describe anticancer benefits of cannabinoids in pediatric cancer patients, formulating a rigorous conclusion on their true effects is not possible. This is because the cannabis products used are varied, ranging from synthetic cannabinoids to whole-plant extracts or cannabinoids purified from plant extracts (purified oils). The exact components of the substances used are not well described, and cannabinoid concentrations within plant extracts have not been comprehensively documented (as would be done in a conventional clinical trial). In addition, the dosage and route of cannabinoid administration differ across reports. To this end, a recent Australian study found significant variability in the actual vs. reported cannabinoid content and profile of extracts purchased via nontraditional means [52]. Moreover, many cancer patients have received conventional therapeutics (such as radiotherapy and chemotherapy) either prior to cannabinoid therapy or concurrently. Thus, to date, there are no studies that comprehensively demonstrate that cannabinoids have antitumoral benefits in childhood brain cancer, yet the anecdotal positive responses that have been reported sustains significant interest in this type of medication.

## 5. Cannabinoid Metabolism and Potential Interactions with Other Cancer Therapeutics

**Figure 3:**
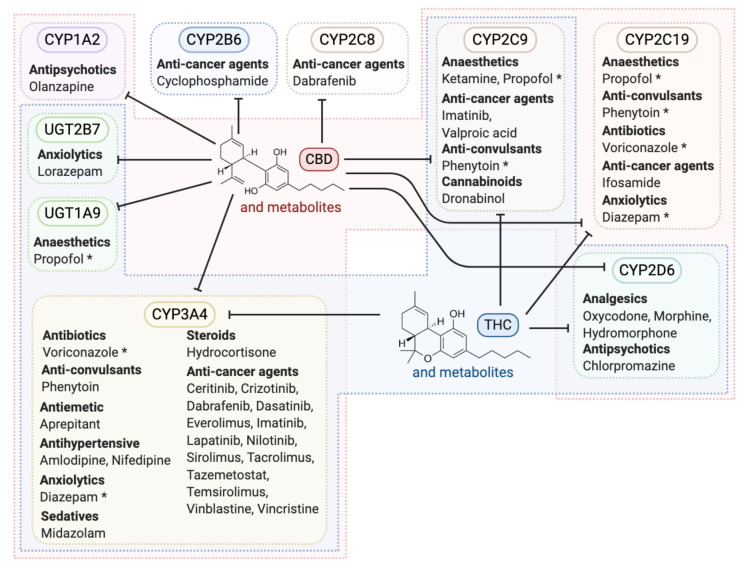
Potential interactions between CBD and/or THC and other drugs used in pediatric oncology. Shown are various drugs used in childhood cancer care that have the potential to be affected by CBD, THC, or their metabolites, divided into groups based on which enzymes they are known to be metabolized by. Note that several drugs are metabolized by more than one enzyme (indicated with an asterisk). Enzymes enclosed in the red shaded area are involved in the metabolism of CBD in humans, while enzymes in the area shaded blue are involved in the metabolism of THC, while no shading indicates enzymes that do not metabolize CBD or THC. The arrows extending from THC and CBD indicate the enzymatic interactions that have been observed, which are mostly inhibitory.

Cannabinoids are predominantly metabolized in the liver by hepatic enzymes such as those in the cytochrome p450 family and glucuronosyltransferases (UGTs) [53], a large family of hepatic enzymes responsible for 75% of all drug metabolism in humans [54]. Specifically, CYP3A4 is primarily involved in the metabolism of THC and CBD [53,55]. Of note, this enzyme is also important in the metabolism of several brain cancer chemotherapeutics, including cyclophosphamide and vincristine [56,57]. Therefore, there is the potential that co-administration of cannabinoids with conventional cancer treatment protocols could alter the bioavailability of chemotherapeutics, prolonging their cytotoxic effects. The liver is also the primary location of metabolism for numerous other drugs, including several pharmaceuticals used in the treatment of cancer, such as anticonvulsants, analgesics and traditional chemotherapies. As such, cannabinoid use during conventional cancer therapy should be very carefully considered. Knowledge of which drugs are metabolized by the same enzymes that metabolize cannabinoids and the inhibitory or inductive effect of cannabinoids on these enzymes will allow for the prediction and understanding of drug-drug interactions (Figure 3 and Table S2). As evidence for potentially detrimental drug interactions, a recent study of 42 epileptic children found almost one-third of those taking concomitant CBD and anticonvulsant valproate recorded abnormal liver function tests and increased aspartate and alanine aminotransferase levels [58]. Whether these effects on liver function are transient and reversible is unknown. Interestingly, the brain and brain stem are an extra-hepatic metabolic site and express several p450 enzymes, including CYP3A4 [59]. As the expression of p450 enzymes in the brain is heterogeneous among individuals [60], this could cause differences for cannabinoid or other drug effects and impact either treatment response or toxicity across individuals.

It is well established that drug metabolism in children differs from adults [61]. While the activity of the main enzyme that metabolizes THC and CBD (CYP3A4) is not significantly different between these two age groups [62], a pharmacokinetic study on synthetic CBD reported some differences in CBD plasma concentrations between infants, children and adults, with higher maximal CBD plasma associated with increasing age [63]. Differences in activity of other THC/CBD metabolizing enzymes, specifically CYP2C19, have been described with higher activity reported in children compared to adults [62]. It has been speculated this may facilitate the higher pediatric clearance of voriconazole compared with adults [62], and as such, may also mediate higher clearance of cannabinoids in children.

## 6. Role for Cannabinoids to Improve Quality of Life for Pediatric Cancer Patients

Instead of directly investigating the anticancer effects of THC and CBD, there are several clinical studies that have aimed to evaluate the role of cannabinoids in symptom management and improvements in quality of life (QOL) for children with cancer. To date, most of our understanding of the utility of cannabinoids in managing symptoms associated with cancer or cancer treatments is derived from adult studies where cannabinoid administration can increase appetite, reduce chemotherapy-induced nausea and vomiting, and improve mood [14,64]. However, these studies are limited by small cohorts and heterogenous cannabinoid products.

There are several historic studies that examined cannabinoids as antiemetic agents in pediatric cancer patients. Two randomized, double-blinded trials showed THC was a superior antiemetic compared to placebo [65,66]. A follow-up study found patients with chemotherapy-induced nausea and vomiting responded better to THC than prochlorperazine, a common anti-sickness medicine [67]. However, the benefits of THC appeared to be specific to the chemotherapy being used for treatment because while THC had antiemetic properties for sarcoma patients on high-dose methotrexate, this was not the case for patients treated with doxorubicin and cyclophosphamide [68]. These studies did not include children with brain cancer. A more recent report describing the experience of patients at Children’s Minnesota, which did include children with brain cancer, states that cannabinoid use in conjunction with traditional antiemetic regimens does improve the patient chemotherapy experience and quality of life [69].

Seizures are a common symptom of brain cancer [70]. Medical cannabis (synthetic or plant-derived extracts) has been investigated in 11 studies for children and adolescents affected by epilepsy [71]. In these noncancer patients, most experienced a reduction in seizures with cannabinoid administration, and while no major side effects were associated with CBD [51], THC was found to cause drowsiness and dizziness that increased in severity at higher doses [71]. There is one report of using CBD for seizure management in an adolescent with brain-tumor related epilepsy. However, they experienced increased seizure frequency, albeit with a reported reduction in seizure severity, while adults in this trial experienced a reduction in seizure frequency [72].

More recently, a single institution in Israel reported their experience over 15 years in 50 pediatric patients (including 9 with brain tumors) where patients were prescribed medical cannabis for cancer and related nausea, vomiting, pain, loss of appetite and depressed mood [73]. The compounds were provided by several producers/distributors licensed by the Israel Ministry of Health and delivered via oral oil drops, inhalation or a combination of both. Questionnaire-based assessments revealed 80% of patients were highly satisfied with cannabinoid treatment, reporting that its usage improved some or most of their cancer-related physical and psychological suffering (domains assessed included pain, nausea and vomiting, sleep, appetite and mood). Reports of negative side effects were infrequent [73]. However, the study is limited by its retrospective approach and the lack of detailed outcomes.

Overall, this and previous studies are important first steps to aid our understanding of these compounds and their benefits, but our ability to form conclusions is limited by the heterogeneity of the cannabinoid products used and differences in route of delivery. Typically, oral administered cannabinoids rarely reach 20% bioavailability [74], while it can be as high as 45% when inhaled [75]. The fact that 60% of patients described by Ofir et al. used the oral route may explain why so few negative side effects were reported [73].

A trial currently being conducted out of the Children’s Hospital Colorado will evaluate the impact of cannabis-derived products for children with CNS tumors who choose to self-medicate (clinicaltrials.gov identifier: NCT03052738). This much-needed study will make use of questionnaires to measure the quality of life and, importantly, will also collect peripheral blood samples to assess blood cannabinoid levels [76]. Such data are essential to determine if there is a true correlation between measurable patient outcomes and cannabinoid exposure.

## 7. Risk of Cannabinoid Use in Children

Although controlled medical cannabis use in adults is reported to be safe and well-tolerated, it should not be assumed that it is safe for children and adolescents. The ECS plays a major role during brain development with CB_1_R, 2-AG and AEA all present in the brain from early in prenatal development [77]. Endocannabinoids influence neurodevelopment by regulating neuronal migration, while CB_1_R has been reported to have roles in neuronal precursor proliferation, migration, axonal elongation, as well as synaptogenesis and myelination later in development (reviewed in [78]). In zebrafish, a common model for research in the ECS, in utero exposure to THC and CBD was shown to cause morphological defects such as shorter body length [79], although the concentrations tested were significantly higher than those achievable in human plasma. Murine studies demonstrate that exogenous cannabinoid exposure during embryogenesis can disrupt neurotransmitter systems resulting in altered motor function and reproductive function, but these studies focused only on THC and also used high concentrations that would not be considered suitable in a pediatric cancer setting [80,81,82].

The critical role of the ECS in mediating neural and cognitive function are not isolated to gestation or early childhood. During adolescence, CB_1_R activation mediates the maturation of interactions between the pre-frontal cortex, amygdala and hippocampus—neural centers responsible for emotion and stress-related behaviors [83]. Furthermore, CB_1_R-mediated processes are involved in the regulation of neurogenesis, memory, learning, cognition, reward centers and depression [84]. It is therefore conceivable that disruption of normal ECS functions by exogenous THC use may alter a range of brain functions. By far, the majority of reports describing cannabinoid effects in adolescents focus on cohorts with self-reported chronic smoked cannabis use where the amount of THC consumed is seldom measured or reported. Inhalation of smoked cannabis can cause short-term physiological effects, such as tachycardia, somnolence and xerostomia, and psychological effects such as paranoia, short-term memory loss and anxiolysis [85]. Prolonged cannabis use via inhalation in adolescents is associated with mental health problems and drug dependence [86]. However, a rigorous longitudinal co-twin control study found that short-term cannabis use had no significant effect on IQ or executive functions, even among heavy cannabis users [87]. The study stated that familial background factors played a major role in predicting whether adolescent cannabis users would perform worse on IQ and executive function tests. The notion that lower IQ precedes cannabis use in adolescents is supported by other longitudinal studies [88,89]. Given the complexity of factors surrounding this multigenerational chicken-or-the-egg conundrum, it is unsurprising that the evidence on the harm of cannabis is conflicting.

A major distinction between THC and CBD is that CBD does not signal via CB_1_R, and in fact, preclinical models have shown that CBD has neuroprotective activity in conditions of oxygen and glucose deprivation [7]. In a review of five clinical trials analyzing CBD treatment in over 1000 pediatric patients suffering from Dravet syndrome, it was found that a daily dose of 20 mg/kg for a period of up to 14 weeks was safe and well-tolerated [90]. Not only did CBD significantly reduce seizure frequency in all trials, but the only adverse effects experienced during administration were somnolence, diarrhea, and decreased appetite. Importantly, no adverse mental or cognitive effects were reported. While these results indicate low CBD toxicity over the period of administration, they are limited due to a lack of long-term cognitive examination.

Whether cannabinoid administration to children or adolescents could cause long-term disruptions in cognition and neurological functioning or exacerbate CNS damage caused by conventional cancer treatments requires further investigation. Furthermore, specifically in the cancer setting, it remains unknown whether cannabinoids can be safely or effectively administered to the pediatric population in combination with other conventional cancer treatments. In the absence of conclusive studies, the American Academy of Pediatrics has taken a cautious view and does not approve of cannabinoid usage in children [91,92].

## 8. Conclusions

The debate over cannabinoid use in children’s cancer continues due to a complete lack of rigorous and thorough literature describing its safety and efficacy. As cannabis is increasingly legalized around the world, there is a greater demand for its utilization in medicine by parents of children with severe diseases and the urgency to elucidate their efficacy and safety intensifies. Current preclinical evidence supports their efficacy and safety in adult brain tumors, with some indications that cannabinoids may interact synergistically with selected chemotherapies. However, this is yet to be demonstrated clinically.

Despite some promising reports, any data demonstrating the potential benefit of cannabinoids for pediatric cancer patients is preliminary. Our knowledge regarding the cellular mechanisms of action for cannabinoids in different cancer types or between adult versus pediatric cancers is still not well understood and presents a challenge when trying to translate results from adult clinical trials to children. Existing studies, unfortunately, employ a broad variety of methodologies to assess the antitumor effects of THC and CBD, as well as differences in the types of cannabinoids (purified from plants versus synthetic), formulations (plant extracts or pure compounds), doses, and routes of administration utilized. Unsurprisingly, this has led to differences in the observed effects and mechanisms of action. Given the important role of the ECS on neural development in early life, the impact of exogenous cannabinoids in children requires careful consideration; however, the realm of research in this area is currently barren. Further research is vital to clarify whether there is true potential for the use of cannabinoids as therapeutic agents in the management of pediatric cancers. Moreover, carefully controlled long-term studies are necessary to determine if medical cannabis use in children does have negative neurodevelopmental consequences and if these outweigh their anti-neoplastic utility.

## Data Availability

No new data were created or analyzed in this study. Data sharing is not applicable to this article.

## Supplementary Materials

The following are available online at https://www.mdpi.com/2072-6694/13/1/157/s1, Table S1. Summary of selected studies that have investigated the anticancer efficacy of THC and/or CBD in preclinical mouse models used to generate Figure 2. Table S2. Table of published sources used to generate Figure 3.

## References

[1] Calvi L., Pentimalli D., Panseri S., Giupponi L., Gelmini F., Beretta G., Vitali D., Bruno M., Zilio E., Pavlovic R. (2018). Comprehensive quality evaluation of medical Cannabis sativa L. inflorescence and macerated oils based on HS-SPME coupled to GC-MS and LC-HRMS (q-exactive orbitrap^®^) approach. J. Pharm. Biomed. Anal..

[2] Lu H.C., Mackie K. (2016). An Introduction to the Endogenous Cannabinoid System. Biol. Psychiatry.

[3] Matsuda L.A., Lolait S.J., Brownstein M.J., Young A.C., Bonner T.I. (1990). Structure of a cannabinoid receptor and functional expression of the cloned cDNA. Nature.

[4] Munro S., Thomas K.L., Abu-Shaar M. (1993). Molecular characterization of a peripheral receptor for cannabinoids. Nature.

[5] Herkenham M., Lynn A.B., Little M.D., Johnson M.R., Melvin L.S., de Costa B.R., Rice K.C. (1990). Cannabinoid receptor localization in brain. Proc. Natl. Acad. Sci. USA.

[6] Atwood B.K., Mackie K. (2010). CB2: A cannabinoid receptor with an identity crisis. Br. J. Pharmacol..

[7] Castillo A., Tolón M.R., Fernández-Ruiz J., Romero J., Martinez-Orgado J. (2010). The neuroprotective effect of cannabidiol in an in vitro model of newborn hypoxic-ischemic brain damage in mice is mediated by CB(2) and adenosine receptors. Neurobiol. Dis..

[8] Pacher P., Kogan N.M., Mechoulam R. (2020). Beyond THC and Endocannabinoids. Annu. Rev. Pharmacol. Toxicol..

[9] Lu H.C., Mackie K. (2020). Review of the Endocannabinoid System. Biol. Psychiatry Cogn. Neurosci. Neuroimaging.

[10] Galve-Roperh I., Chiurchiù V., Díaz-Alonso J., Bari M., Guzmán M., Maccarrone M. (2013). Cannabinoid receptor signaling in progenitor/stem cell proliferation and differentiation. Prog. Lipid Res..

[11] Velasco G., Sánchez C., Guzmán M. (2016). Anticancer mechanisms of cannabinoids. Curr. Oncol..

[12] Braun I.M., Wright A., Peteet J., Meyer F.L., Yuppa D.P., Bolcic-Jankovic D., LeBlanc J., Chang Y., Yu L., Nayak M.M. (2018). Medical Oncologists’ Beliefs, Practices, and Knowledge Regarding Marijuana Used Therapeutically: A Nationally Representative Survey Study. J. Clin. Oncol..

[13] Zylla D., Steele G., Eklund J., Mettner J., Arneson T. (2018). Oncology Clinicians and the Minnesota Medical Cannabis Program: A Survey on Medical Cannabis Practice Patterns, Barriers to Enrollment, and Educational Needs. Cannabis Cannabinoid Res..

[14] Wilkie G., Sakr B., Rizack T. (2016). Medical Marijuana Use in Oncology: A Review. JAMA Oncol..

[15] Adler J.N., Colbert J.A. (2013). Clinical decisions. Medicinal use of marijuana--polling results. N. Engl. J. Med..

[16] Ryan J.E., Smeltzer S.C., Sharts-Hopko N.C. (2020). Parents’ experiences using medical cannabis for their child. Nurs. Outlook.

[17] Ananth P., Reed-Weston A., Wolfe J. (2018). Medical marijuana in pediatric oncology: A review of the evidence and implications for practice. Pediatr. Blood Cancer.

[18] Ramer R., Hinz B. (2017). Cannabinoids as Anticancer Drugs. Adv. Pharmacol..

[19] Lombard C., Nagarkatti M., Nagarkatti P.S. (2005). Targeting cannabinoid receptors to treat leukemia: Role of cross-talk between extrinsic and intrinsic pathways in Delta9-tetrahydrocannabinol (THC)-induced apoptosis of Jurkat cells. Leuk. Res..

[20] Olivas-Aguirre M., Torres-Lopez L., Valle-Reyes J.S., Hernandez-Cruz A., Pottosin I., Dobrovinskaya O. (2019). Cannabidiol directly targets mitochondria and disturbs calcium homeostasis in acute lymphoblastic leukemia. Cell Death Dis..

[21] Jia W., Hegde V.L., Singh N.P., Sisco D., Grant S., Nagarkatti M., Nagarkatti P.S. (2006). Delta9-tetrahydrocannabinol-induced apoptosis in Jurkat leukemia T cells is regulated by translocation of Bad to mitochondria. Mol. Cancer Res..

[22] Herrera B., Carracedo A., Diez-Zaera M., Gómez del Pulgar T., Guzmán M., Velasco G. (2006). The CB2 cannabinoid receptor signals apoptosis via ceramide-dependent activation of the mitochondrial intrinsic pathway. Exp. Cell Res..

[23] Soto-Mercado V., Mendivil-Perez M., Jimenez-Del-Rio M., Fox J.E., Velez-Pardo C. (2020). Cannabinoid CP55940 selectively induces apoptosis in Jurkat cells and in ex vivo T-cell acute lymphoblastic leukemia through H(2)O(2) signaling mechanism. Leuk. Res..

[24] McKallip R.J., Jia W., Schlomer J., Warren J.W., Nagarkatti P.S., Nagarkatti M. (2006). Cannabidiol-induced apoptosis in human leukemia cells: A novel role of cannabidiol in the regulation of p22phox and Nox4 expression. Mol. Pharmacol..

[25] Hernández-Tiedra S., Fabriàs G., Dávila D., Salanueva Í.J., Casas J., Montes L.R., Antón Z., García-Taboada E., Salazar-Roa M., Lorente M. (2016). Dihydroceramide accumulation mediates cytotoxic autophagy of cancer cells via autolysosome destabilization. Autophagy.

[26] Seltzer E.S., Watters A.K., MacKenzie D., Granat L.M., Zhang D. (2020). Cannabidiol (CBD) as a Promising Anti-Cancer Drug. Cancers.

[27] Kalenderoglou N., Macpherson T., Wright K.L. (2017). Cannabidiol Reduces Leukemic Cell Size—But Is It Important?. Front. Pharmacol..

[28] Salazar M., Carracedo A., Salanueva Í.J., Hernández-Tiedra S., Lorente M., Egia A., Vázquez P., Blázquez C., Torres S., García S. (2009). Cannabinoid action induces autophagy-mediated cell death through stimulation of ER stress in human glioma cells. J. Clin. Investig..

[29] Vara D., Salazar M., Olea-Herrero N., Guzmán M., Velasco G., Díaz-Laviada I. (2011). Anti-tumoral action of cannabinoids on hepatocellular carcinoma: Role of AMPK-dependent activation of autophagy. Cell Death Differ..

[30] Liu W.M., Scott K.A., Shamash J., Joel S., Powles T.B. (2008). Enhancing the in vitro cytotoxic activity of Delta9-tetrahydrocannabinol in leukemic cells through a combinatorial approach. Leuk. Lymphoma.

[31] Scott K.A., Dalgleish A.G., Liu W.M. (2017). Anticancer effects of phytocannabinoids used with chemotherapy in leukaemia cells can be improved by altering the sequence of their administration. Int. J. Oncol..

[32] Oesch S., Walter D., Wachtel M., Pretre K., Salazar M., Guzman M., Velasco G., Schafer B.W. (2009). Cannabinoid receptor 1 is a potential drug target for treatment of translocation-positive rhabdomyosarcoma. Mol. Cancer Ther..

[33] Carracedo A., Gironella M., Lorente M., Garcia S., Guzmán M., Velasco G., Iovanna J.L. (2006). Cannabinoids induce apoptosis of pancreatic tumor cells via endoplasmic reticulum stress-related genes. Cancer Res..

[34] Notaro A., Sabella S., Pellerito O., Di Fiore R., De Blasio A., Vento R., Calvaruso G., Giuliano M. (2014). Involvement of PAR-4 in cannabinoid-dependent sensitization of osteosarcoma cells to TRAIL-induced apoptosis. Int. J. Biol. Sci..

[35] Fisher T., Golan H., Schiby G., PriChen S., Smoum R., Moshe I., Peshes-Yaloz N., Castiel A., Waldman D., Gallily R. (2016). In vitro and in vivo efficacy of non-psychoactive cannabidiol in neuroblastoma. Curr. Oncol..

[36] Ma X., Liu Y., Liu Y., Alexandrov L.B., Edmonson M.N., Gawad C., Zhou X., Li Y., Rusch M.C., Easton J. (2018). Pan-cancer genome and transcriptome analyses of 1,699 paediatric leukaemias and solid tumours. Nature.

[37] Borrell B. (2010). How accurate are cancer cell lines?. Nature.

[38] Siegel R.L., Miller K.D., Jemal A. (2020). Cancer statistics, 2020. CA A Cancer J. Clin..

[39] López-Valero I., Saiz-Ladera C., Torres S., Hernández-Tiedra S., García-Taboada E., Rodríguez-Fornés F., Barba M., Dávila D., Salvador-Tormo N., Guzmán M. (2018). Targeting Glioma Initiating Cells with A combined therapy of cannabinoids and temozolomide. Biochem. Pharmacol..

[40] Torres S., Lorente M., Rodríguez-Fornés F., Hernández-Tiedra S., Salazar M., García-Taboada E., Barcia J., Guzmán M., Velasco G. (2011). A combined preclinical therapy of cannabinoids and temozolomide against glioma. Mol. Cancer Ther..

[41] Scott K.A., Dalgleish A.G., Liu W.M. (2014). The combination of cannabidiol and Delta9-tetrahydrocannabinol enhances the anticancer effects of radiation in an orthotopic murine glioma model. Mol. Cancer Ther..

[42] Ellert-Miklaszewska A., Grajkowska W., Gabrusiewicz K., Kaminska B., Konarska L. (2007). Distinctive pattern of cannabinoid receptor type II (CB2) expression in adult and pediatric brain tumors. Brain Res..

[43] Sredni S.T., Huang C.C., Suzuki M., Pundy T., Chou P., Tomita T. (2016). Spontaneous involution of pediatric low-grade gliomas: High expression of cannabinoid receptor 1 (CNR1) at the time of diagnosis may indicate involvement of the endocannabinoid system. Childs Nerv. Syst..

[44] Taylor M.D., Poppleton H., Fuller C., Su X., Liu Y., Jensen P., Magdaleno S., Dalton J., Calabrese C., Board J. (2005). Radial glia cells are candidate stem cells of ependymoma. Cancer Cell.

[45] Vladoiu M.C., El-Hamamy I., Donovan L.K., Farooq H., Holgado B.L., Sundaravadanam Y., Ramaswamy V., Hendrikse L.D., Kumar S., Mack S.C. (2019). Childhood cerebellar tumours mirror conserved fetal transcriptional programs. Nature.

[46] Petersen G., Moesgaard B., Schmid P.C., Schmid H.H., Broholm H., Kosteljanetz M., Hansen H.S. (2005). Endocannabinoid metabolism in human glioblastomas and meningiomas compared to human non-tumour brain tissue. J. Neurochem..

[47] Wu X., Han L., Zhang X., Li L., Jiang C., Qiu Y., Huang R., Xie B., Lin Z., Ren J. (2012). Alteration of endocannabinoid system in human gliomas. J. Neurochem..

[48] Maccarrone M., Attinà M., Cartoni A., Bari M., Finazzi-Agrò A. (2001). Gas chromatography-mass spectrometry analysis of endogenous cannabinoids in healthy and tumoral human brain and human cells in culture. J. Neurochem..

[49] De Jesús M.L., Hostalot C., Garibi J.M., Sallés J., Meana J.J., Callado L.F. (2010). Opposite changes in cannabinoid CB1 and CB2 receptor expression in human gliomas. Neurochem. Int..

[50] Foroughi M., Hendson G., Sargent M.A., Steinbok P. (2011). Spontaneous regression of septum pellucidum/forniceal pilocytic astrocytomas--possible role of Cannabis inhalation. Childs Nerv. Syst..

[51] Kenyon J., Liu W., Dalgleish A. (2018). Report of Objective Clinical Responses of Cancer Patients to Pharmaceutical-grade Synthetic Cannabidiol. Anticancer Res..

[52] Suraev A., Lintzeris N., Stuart J., Kevin R.C., Blackburn R., Richards E., Arnold J.C., Ireland C., Todd L., Allsop D.J. (2018). Composition and Use of Cannabis Extracts for Childhood Epilepsy in the Australian Community. Sci. Rep..

[53] Matsunaga T., Iwawaki Y., Watanabe K., Yamamoto I., Kageyama T., Yoshimura H. (1995). Metabolism of delta 9-tetrahydrocannabinol by cytochrome P450 isozymes purified from hepatic microsomes of monkeys. Life Sci..

[54] Guengerich F.P. (2008). Cytochrome p450 and chemical toxicology. Chem. Res. Toxicol..

[55] Bergamaschi M.M., Queiroz R.H., Zuardi A.W., Crippa J.A. (2011). Safety and side effects of cannabidiol, a Cannabis sativa constituent. Curr. Drug Saf..

[56] Huang Z., Roy P., Waxman D.J. (2000). Role of human liver microsomal CYP3A4 and CYP2B6 in catalyzing N-dechloroethylation of cyclophosphamide and ifosfamide. Biochem. Pharmacol..

[57] Baumhäkel M., Kasel D., Rao-Schymanski R.A., Böcker R., Beckurts K.T., Zaigler M., Barthold D., Fuhr U. (2001). Screening for inhibitory effects of antineoplastic agents on CYP3A4 in human liver microsomes. Int. J. Clin. Pharmacol. Ther..

[58] Gaston T.E., Bebin E.M., Cutter G.R., Liu Y., Szaflarski J.P., Program U.C. (2017). Interactions between cannabidiol and commonly used antiepileptic drugs. Epilepsia.

[59] Krishna D.R., Klotz U. (1994). Extrahepatic metabolism of drugs in humans. Clin. Pharm..

[60] Ferguson C.S., Tyndale R.F. (2011). Cytochrome P450 enzymes in the brain: Emerging evidence of biological significance. Trends Pharmacol. Sci..

[61] McLeod H.L., Relling M.V., Crom W.R., Silverstein K., Groom S., Rodman J.H., Rivera G.K., Crist W.M., Evans W.E. (1992). Disposition of antineoplastic agents in the very young child. Br. J. Cancer Suppl..

[62] Zane N.R., Chen Y., Wang M.Z., Thakker D.R. (2018). Cytochrome P450 and flavin-containing monooxygenase families: Age-dependent differences in expression and functional activity. Pediatr. Res..

[63] Wheless J.W., Dlugos D., Miller I., Oh D.A., Parikh N., Phillips S., Renfroe J.B., Roberts C.M., Saeed I., Sparagana S.P. (2019). Pharmacokinetics and Tolerability of Multiple Doses of Pharmaceutical-Grade Synthetic Cannabidiol in Pediatric Patients with Treatment-Resistant Epilepsy. CNS Drugs.

[64] Whiting P.F., Wolff R.F., Deshpande S., Di Nisio M., Duffy S., Hernandez A.V., Keurentjes J.C., Lang S., Misso K., Ryder S. (2015). Cannabinoids for Medical Use: A Systematic Review and Meta-analysis. JAMA.

[65] Sallan S.E., Zinberg N.E., Frei E. (1975). Antiemetic effect of delta-9-tetrahydrocannabinol in patients receiving cancer chemotherapy. N. Engl. J. Med..

[66] Chang A.E., Shiling D.J., Stillman R.C., Goldberg N.H., Seipp C.A., Barofsky I., Simon R.M., Rosenberg S.A. (1979). Delata-9-tetrahydrocannabinol as an antiemetic in cancer patients receiving high-dose methotrexate. A prospective, randomized evaluation. Ann. Intern. Med..

[67] Sallan S.E., Cronin C., Zelen M., Zinberg N.E. (1980). Antiemetics in patients receiving chemotherapy for cancer: A randomized comparison of delta-9-tetrahydrocannabinol and prochlorperazine. N. Engl. J. Med..

[68] Chang A.E., Shiling D.J., Stillman R.C., Goldberg N.H., Seipp C.A., Barofsky I., Rosenberg S.A. (1981). A prospective evaluation of delta-9-tetrahydrocannabinol as an antiemetic in patients receiving adriamycin and cytoxan chemotherapy. Cancer.

[69] Skrypek M.M., Bostrom B.C., Bendel A.E. (2019). Medical Cannabis Certification in a Large Pediatric Oncology Center. Children.

[70] Udaka Y.T., Packer R.J. (2018). Pediatric Brain Tumors. Neurol. Clin..

[71] Wong S.S., Wilens T.E. (2017). Medical Cannabinoids in Children and Adolescents: A Systematic Review. Pediatrics.

[72] Warren P.P., Bebin E.M., Nabors L.B., Szaflarski J.P. (2017). The use of cannabidiol for seizure management in patients with brain tumor-related epilepsy. Neurocase.

[73] Ofir R., Bar-Sela G., Weyl Ben-Arush M., Postovsky S. (2019). Medical marijuana use for pediatric oncology patients: Single institution experience. Pediatr. Hematol. Oncol..

[74] Borgelt L.M., Franson K.L., Nussbaum A.M., Wang G.S. (2013). The pharmacologic and clinical effects of medical cannabis. Pharmacotherapy.

[75] Huestis M.A. (2007). Human cannabinoid pharmacokinetics. Chem. Biodivers.

[76] Dorris K., Channell J., Hemenway M., Baroffio A., Ellison M., Brionse N., Griesinger A., Donson A., Madden J., Van Essen C. (2018). QOL-52. Use of Cannabinoids in the Pediatric Central Nervous System Tumor Population. Neuro-Oncol..

[77] Meyer H.C., Lee F.S., Gee D.G. (2018). The Role of the Endocannabinoid System and Genetic Variation in Adolescent Brain Development. Neuropsychopharmacology.

[78] Harkany T., Guzmán M., Galve-Roperh I., Berghuis P., Devi L.A., Mackie K. (2007). The emerging functions of endocannabinoid signaling during CNS development. Trends Pharmacol. Sci..

[79] Ahmed K.T., Amin M.R., Shah P., Ali D.W. (2018). Motor neuron development in zebrafish is altered by brief (5-hr) exposures to THC ((9)-tetrahydrocannabinol) or CBD (cannabidiol) during gastrulation. Sci. Rep..

[80] Dalterio S.L. (1980). Perinatal or adult exposure to cannabinoids alters male reproductive functions in mice. Pharmacol. Biochem. Behav..

[81] Navarro M., Rodríguez de Fonseca F., Hernández M.L., Ramos J.A., Fernández-Ruiz J.J. (1994). Motor behavior and nigrostriatal dopaminergic activity in adult rats perinatally exposed to cannabinoids. Pharmacol. Biochem. Behav..

[82] de Salas-Quiroga A., Díaz-Alonso J., García-Rincón D., Remmers F., Vega D., Gómez-Cañas M., Lutz B., Guzmán M., Galve-Roperh I. (2015). Prenatal exposure to cannabinoids evokes long-lasting functional alterations by targeting CB1 receptors on developing cortical neurons. Proc. Natl. Acad. Sci. USA.

[83] Dow-Edwards D., Silva L. (2017). Endocannabinoids in brain plasticity: Cortical maturation, HPA axis function and behavior. Brain Res..

[84] Mechoulam R., Parker L.A. (2013). The endocannabinoid system and the brain. Annu. Rev. Psychol..

[85] Hadland S.E., Harris S.K. (2014). Youth marijuana use: State of the science for the practicing clinician. Curr. Opin. Pediatr..

[86] Volkow N.D., Baler R.D., Compton W.M., Weiss S.R. (2014). Adverse health effects of marijuana use. N. Engl. J. Med..

[87] Meier M.H., Caspi A., Danese A., Fisher H.L., Houts R., Arseneault L., Moffitt T.E. (2018). Associations between adolescent cannabis use and neuropsychological decline: A longitudinal co-twin control study. Addiction.

[88] Jackson N.J., Isen J.D., Khoddam R., Irons D., Tuvblad C., Iacono W.G., McGue M., Raine A., Baker L.A. (2016). Impact of adolescent marijuana use on intelligence: Results from two longitudinal twin studies. Proc. Natl. Acad. Sci. USA.

[89] Castellanos-Ryan N., Pingault J.B., Parent S., Vitaro F., Tremblay R.E., Seguin J.R. (2017). Adolescent cannabis use, change in neurocognitive function, and high-school graduation: A longitudinal study from early adolescence to young adulthood. Dev. Psychopathol..

[90] Nabbout R., Thiele E.A. (2020). The role of cannabinoids in epilepsy treatment: A critical review of efficacy results from clinical trials. Epileptic Disord..

[91] Golan H., Fisher T., Toren A. (2017). The Role of Cannabinoids in the Treatment of Cancer in Pediatric Patients. ISR Med. Assoc. J..

[92] Ammerman S., Ryan S., Adelman W.P. (2015). Committee on Substance Abuse, t.C.o.A. The impact of marijuana policies on youth: Clinical, research, and legal update. Pediatrics.

