# Supplementary material for: The Role of Cannabinoids as Anticancer Agents in Pediatric Oncology
Source: Cancers (Basel). 2021 Jan 5;13(1):157. doi: 10.3390/cancers13010157 (PMC7796497; doi:10.3390/cancers13010157)
Supplement: Supplementary file 1 [file cancers-13-00157-s001.pdf]

Supplementary Material: The Role of Cannabinoids as Anti-Cancer Agents in Paediatric Oncology

Clara Andradas, Alexandra Truong, Jacob Byrne and Raelene Endersby

Table S1. Summary of selected studies that have investigated the anti-cancer efficacy of THC and/or CBD in pre-clinical mouse models used to generate Figure 2. Colours correspond to the symbols in Figure 2.

Model type		Cancer type	Compound Tested	References
Immune-Competent Models	Ectopic	Lung carcinoma	THC	[7]
		Breast carcinoma	THC	[8]
		Lymphoma	THC	[9]
		Melanoma	CBD	[10]
		Leukaemia (paediatric)	CBD	[1]
	Orthotopic	Breast carcinoma	CBD	[11]
		Melanoma	THC	[12]
		Colorectal (carcinogen-induced tumours)	CBD	[13]
Immune-Deficient Models	Ectopic	Melanoma	THC, CBD	[14]
		Colorectal cancer	CBD	[15–17]
		Prostate cancer	CBD	[18]
		Gastric cancer	CBD	[19]
		Glioblastoma	THC, CBD	[20,21]
		Lung cancer	THC, CBD	[22,23]
		Hepatocellular carcinoma	THC	[2]
		Pancreatic cancer	THC	[4]
		Neuroblastoma	THC, CBD	[5]
		Rhabdomyosarcoma	THC	[3]
	Orthotopic	Breast cancer	THC, CBD	[8,11]
		Glioblastoma	THC, CBD	[6,21]
		Hepatocellular carcinoma	THC	[2]
		Pancreatic cancer	THC	[4]
Genetically Modified Mice		Breast cancer	THC	[8]
		Pancreatic cancer	CBD	[24]

Table S2. Table of published sources used to generate Figure 3. Shown are drug metabolising enzymes reported to be affected by THC and CBD, with a list of other drugs used in childhood cancer treatment that are also affected by those enzymes. The reported effects of each cannabinoid are shown with matching citations which have been crosschecked with [106].

Enzyme	Possible Drug Interactions		Reported Effect of Cannabinoids on Each Enzyme	
			CBD	THC
CYP1A2	Antipsychotics	Olanzapine	• Substrate [26]<br>• Inhibits [27]	• Likely inhibitor [28]<br>• Possible inducer [27,29]
CYP2B6	Anti-cancer agents	Cyclophosphamide	• Inhibits [27]	• Inhibits [27]
CYP2C8	Anti-cancer agents	Dabrafenib	• Inhibits [27]	• n/a
CYP2C9	Anaesthetics<br>Anti-cancer agents:<br>Anticonvulsants<br>Cannabinoids	Ketamine, Propofol<br>Imatinib<br>Phenytoin<br>Dronabinol	• Substrate [26]<br>• Inhibits [27]	• Substrate [30]<br>• Inhibits [27]
CYP2C19	Anaesthetics<br>Antibiotics<br>Anti-cancer agents<br>Anticonvulsants<br>Anxiolytics	Propofol<br>Voriconazole<br>Ifosamide<br>Phenytoin<br>Diazepam	• Substrate [26]<br>• Inhibits [27]	• Substrate [31]<br>• potential inhibitor [28]
CYP2D6	Analgesics:<br>Antipsychotics:	Hydromorphone, Morphine, Oxycodone<br>Chlorpromazine	• Substrate [26]<br>• Inhibits [27]	• Substrate [29]<br>• Inhibits [27]
CYP3A4	Antibiotics<br>Anti-cancer agents<br>Anticonvulsants<br>Antiemetic<br>Anxiolytics<br>Sedatives<br>Steroids	Voriconazole<br>Ceritinib, Crizotinib, Dabrafenib, Dasatinib, Everolimus, Imatinib, Lapatinib, Nilotinib, Sirolimus, Tacrolimus, Tazemetostat, Temsirolimus, Vinblastine, Vincristine<br>Phenytoin<br>Aprepitant<br>Diazepam<br>Midazolam<br>Hydrocortisone	• Substrate [26]<br>• Inhibits [27]	• Metabolites are substrates [29]<br>• Inhibits [27]
UGT1A9	Anaesthetics	Propofol	• Inhibits [27]	• Metabolites are substrates [29]<br>• Inhibited by CBN (THC metabolite) [28]
UGT2B7	Anxiolytics	Lorazepam	• Inhibits [27]	• Metabolites are substrates [111]<br>• Inhibited by CBN (THC metabolite) [28]

References

1. Soto-Mercado, V.; Mendivil-Perez, M.; Jimenez-Del-Rio, M.; Fox, J.E.; Velez-Pardo, C. Cannabinoid CP55940 selectively induces apoptosis in Jurkat cells and in ex vivo T-cell acute lymphoblastic leukemia through H(2)O(2) signaling mechanism. *Leuk. Res.* **2020**, *95*, 106389, doi:10.1016/j.leukres.2020.106389
2. Salazar, M.; Carracedo, A.; Salanueva, Í.J.; Hernández-Tiedra, S.; Lorente, M.; Egia, A.; Vázquez, P.; Blázquez, C.; Torres, S.; García, S.; et al. Cannabinoid action induces autophagy-mediated cell death through stimulation of ER stress in human glioma cells. *J. Clin.Investig.* **2009**, *119*, 1359–1372, doi:10.1172/JCI37948.
3. Scott, K.A.; Dalgleish, A.G.; Liu, W.M. Anticancer effects of phytocannabinoids used with chemotherapy in leukaemia cells can be improved by altering the sequence of their administration. *Int. J. Oncol.* **2017**, *51*, 369–377, doi:10.3892/ijo.2017.4022.
4. Oesch, S.; Walter, D.; Wachtel, M.; Pretre, K.; Salazar, M.; Guzman, M.; Velasco, G.; Schafer, B.W. Cannabinoid receptor 1 is a potential drug target for treatment of translocation-positive rhabdomyosarcoma. *Mol. Cancer Ther.* **2009**, *8*, 1838–1845, doi:10.1158/1535-7163.MCT-08-1147.
5. Notaro, A.; Sabella, S.; Pellerito, O.; Di Fiore, R.; De Blasio, A.; Vento, R.; Calvaruso, G.; Giuliano, M. Involvement of PAR-4 in cannabinoid-dependent sensitization of osteosarcoma cells to TRAIL-induced apoptosis. *Int. J. Biol. Sci.* **2014**, *10*, 466–478, doi:10.7150/ijbs.8337.
6. 39. López-Valero, I.; Saiz-Ladera, C.; Torres, S.; Hernández-Tiedra, S.; García-Taboada, E.; Rodríguez-Fornés, F.; Barba, M.; Dávila, D.; Salvador-Tormo, N.; Guzmán, M.; et al. Targeting Glioma Initiating Cells with A combined therapy of cannabinoids and temozolomide. *Biochem. Pharmacol.* **2018**, *157*, 266–274, doi:10.1016/j.bcp.2018.09.007.
7. Jackson, N.J.; Isen, J.D.; Khoddam, R.; Irons, D.; Tuvblad, C.; Iacono, W.G.; McGue, M.; Raine, A.; Baker, L.A. Impact of adolescent marijuana use on intelligence: Results from two longitudinal twin studies. *Proc. Natl. Acad. Sci. USA* **2016**, *113*, E500-508, doi:10.1073/pnas.1516648113.
8. Castellanos-Ryan, N.; Pingault, J.B.; Parent, S.; Vitaro, F.; Tremblay, R.E.; Seguin, J.R. Adolescent cannabis use, change in neurocognitive function, and high-school graduation: A longitudinal study from early adolescence to young adulthood. *Dev. Psychopathol.* **2017**, *29*, 1253–1266, doi:10.1017/S0954579416001280.
9. Nabbout, R.; Thiele, E.A. The role of cannabinoids in epilepsy treatment: A critical review of efficacy results from clinical trials. *Epileptic Disord.* **2020**, *22*, 23–28, doi:10.1684/epd.2019.1124.
10. Golan, H.; Fisher, T.; Toren, A. The Role of Cannabinoids in the Treatment of Cancer in Pediatric Patients. *ISR Med. Assoc. J.* **2017**, *19*, 89–94.
11. Ammerman, S.; Ryan, S.; Adelman, W.P. Committee on Substance Abuse, t.C.o.A. The impact of marijuana policies on youth: Clinical, research, and legal update. *Pediatrics* **2015**, *135*, e769–e785, doi:10.1542/peds.2014-4147.
12. Munson, A.E.; Harris, L.S.; Friedman, M.A.; Dewey, W.L.; Carchman, R.A. Antineoplastic Activity of Cannabinoids2. *JNCI: Journal of the National Cancer Institute* **1975**, *55*, 597-602, doi:10.1093/jnci/55.3.597.
13. Caffarel, M.M.; Andradas, C.; Mira, E.; Pérez-Gómez, E.; Cerutti, C.; Moreno-Bueno, G.; Flores, J.M.; García-Real, I.; Palacios, J.; Mañes, S., et al. Cannabinoids reduce ErbB2-driven breast cancer progression through Akt inhibition. *Molecular Cancer* **2010**, *9*, 196, doi:10.1186/1476-4598-9-196.
14. McKallip, R.J.; Lombard, C.; Fisher, M.; Martin, B.R.; Ryu, S.; Grant, S.; Nagarkatti, P.S.; Nagarkatti, M. Targeting CB2 cannabinoid receptors as a novel therapy to treat malignant lymphoblastic disease. *Blood* **2002**, *100*, 627-634, doi:10.1182/blood-2002-01-0098.
15. Simmerman, E.; Qin, X.; Yu, J.C.; Baban, B. Cannabinoids as a Potential New and Novel Treatment for Melanoma: A Pilot Study in a Murine Model. *J Surg Res* **2019**, *235*, 210-215, doi:10.1016/j.jss.2018.08.055.
16. McAllister, S.D.; Murase, R.; Christian, R.T.; Lau, D.; Zielinski, A.J.; Allison, J.; Almanza, C.; Pakdel, A.; Lee, J.; Limbad, C., et al. Pathways mediating the effects of cannabidiol on the reduction of breast cancer cell proliferation, invasion, and metastasis. *Breast Cancer Res Treat* **2011**, *129*, 37-47, doi:10.1007/s10549-010-1177-4.
17. Glodde, N.; Jakobs, M.; Bald, T.; Tüting, T.; Gaffal, E. Differential role of cannabinoids in the pathogenesis of skin cancer. *Life Sci* **2015**, *138*, 35-40, doi:10.1016/j.lfs.2015.04.003.
18. Aviello, G.; Romano, B.; Borrelli, F.; Capasso, R.; Gallo, L.; Piscitelli, F.; Di Marzo, V.; Izzo, A.A. Chemopreventive effect of the non-psychotropic phytocannabinoid cannabidiol on experimental colon cancer. *J Mol Med (Berl)* **2012**, *90*, 925-934, doi:10.1007/s00109-011-0856-x.
19. Armstrong, J.L.; Hill, D.S.; McKee, C.S.; Hernandez-Tiedra, S.; Lorente, M.; Lopez-Valero, I.; Eleni Anagnostou, M.; Babatunde, F.; Corazzari, M.; Redfern, C.P.F., et al. Exploiting cannabinoid-induced cytotoxic autophagy to drive melanoma cell death. *J Invest Dermatol* **2015**, *135*, 1629-1637, doi:10.1038/jid.2015.45.
20. Jeong, S.; Kim, B.G.; Kim, D.Y.; Kim, B.R.; Kim, J.L.; Park, S.H.; Na, Y.J.; Jo, M.J.; Yun, H.K.; Jeong, Y.A., et al. Cannabidiol Overcomes Oxaliplatin Resistance by Enhancing NOS3- and SOD2-Induced Autophagy in Human Colorectal Cancer Cells. *Cancers (Basel)* **2019**, *11*, doi:10.3390/cancers11060781.

21. Jeong, S.; Yun, H.K.; Jeong, Y.A.; Jo, M.J.; Kang, S.H.; Kim, J.L.; Kim, D.Y.; Park, S.H.; Kim, B.R.; Na, Y.J., et al. Cannabidiol-induced apoptosis is mediated by activation of Noxa in human colorectal cancer cells. *Cancer Lett* **2019**, *447*, 12-23, doi:10.1016/j.canlet.2019.01.011.
22. Kim, J.L.; Kim, B.R.; Kim, D.Y.; Jeong, Y.A.; Jeong, S.; Na, Y.J.; Park, S.H.; Yun, H.K.; Jo, M.J.; Kim, B.G., et al. Cannabidiol Enhances the Therapeutic Effects of TRAIL by Upregulating DR5 in Colorectal Cancer. *Cancers (Basel)* **2019**, *11*, doi:10.3390/cancers11050642.
23. De Petrocellis, L.; Ligresti, A.; Schiano Moriello, A.; Iappelli, M.; Verde, R.; Stott, C.G.; Cristino, L.; Orlando, P.; Di Marzo, V. Non-THC cannabinoids inhibit prostate carcinoma growth in vitro and in vivo: pro-apoptotic effects and underlying mechanisms. *Br J Pharmacol* **2013**, *168*, 79-102, doi:10.1111/j.1476-5381.2012.02027.x.
24. Jeong, S.; Jo, M.J.; Yun, H.K.; Kim, D.Y.; Kim, B.R.; Kim, J.L.; Park, S.H.; Na, Y.J.; Jeong, Y.A.; Kim, B.G., et al. Cannabidiol promotes apoptosis via regulation of XIAP/Smac in gastric cancer. *Cell Death Dis* **2019**, *10*, 846, doi:10.1038/s41419-019-2001-7.
25. Moreno, E.; Andradas, C.; Medrano, M.; Caffarel, M.M.; Pérez-Gómez, E.; Blasco-Benito, S.; Gómez-Cañas, M.; Pazos, M.R.; Irving, A.J.; Lluís, C., et al. Targeting CB2-GPR55 receptor heteromers modulates cancer cell signaling. *J Biol Chem* **2014**, *289*, 21960-21972, doi:10.1074/jbc.M114.561761.
26. Velasco, G.; Sánchez, C.; Guzmán, M. Towards the use of cannabinoids as antitumour agents. *Nat Rev Cancer* **2012**, *12*, 436-444, doi:10.1038/nrc3247.
27. Preet, A.; Ganju, R.K.; Groopman, J.E. Δ9-Tetrahydrocannabinol inhibits epithelial growth factor-induced lung cancer cell migration in vitro as well as its growth and metastasis in vivo. *Oncogene* **2008**, *27*, 339-346, doi:10.1038/sj.onc.1210641.
28. Ramer, R.; Rohde, A.; Merkord, J.; Rohde, H.; Hinz, B. Decrease of plasminogen activator inhibitor-1 may contribute to the anti-invasive action of cannabidiol on human lung cancer cells. *Pharm Res* **2010**, *27*, 2162-2174, doi:10.1007/s11095-010-0219-2.
29. Ferro, R.; Adamska, A.; Lattanzio, R.; Mavrommati, I.; Edling, C.E.; Arifin, S.A.; Fyffe, C.A.; Sala, G.; Sacchetto, L.; Chiorino, G., et al. GPR55 signalling promotes proliferation of pancreatic cancer cells and tumour growth in mice, and its inhibition increases effects of gemcitabine. *Oncogene* **2018**, *37*, 6368-6382, doi:10.1038/s41388-018-0390-1.
30. Stout, S.M.; Cimino, N.M. Exogenous cannabinoids as substrates, inhibitors, and inducers of human drug metabolizing enzymes: a systematic review. *Drug Metab Rev* **2014**, *46*, 86-95, doi:10.3109/03602532.2013.849268.
31. Jiang, R.; Yamaori, S.; Takeda, S.; Yamamoto, I.; Watanabe, K. Identification of cytochrome P450 enzymes responsible for metabolism of cannabidiol by human liver microsomes. *Life Sci* **2011**, *89*, 165-170, doi:10.1016/j.lfs.2011.05.018.
32. Alsherbiny, M.A.; Li, C.G. Medicinal Cannabis-Potential Drug Interactions. *Medicines (Basel)* **2018**, *6*, 3, doi:10.3390/medicines6010003.
33. Qian, Y.; Gurley, B.J.; Markowitz, J.S. The Potential for Pharmacokinetic Interactions Between Cannabis Products and Conventional Medications. *Journal of Clinical Psychopharmacology* **2019**, *39*, 462-471, doi:10.1097/jcp.0000000000001089.
34. Anderson, G.D.; Chan, L.N. Pharmacokinetic Drug Interactions with Tobacco, Cannabinoids and Smoking Cessation Products. *Clin Pharmacokinet* **2016**, *55*, 1353-1368, doi:10.1007/s40262-016-0400-9.
35. Patilea-Vrana, G.I.; Unadkat, J.D. Quantifying hepatic enzyme kinetics of (-)-Δ9-tetrahydrocannabinol (THC) and its psychoactive metabolite, 11-OH-THC, through in vitro modeling. *Drug Metabolism and Disposition* **2019**, 10.1124/dmd.119.086470, dmd.119.086470, doi:10.1124/dmd.119.086470.
36. Patilea-Vrana, G.I.; Anoshchenko, O.; Unadkat, J.D. Hepatic Enzymes Relevant to the Disposition of (-)-Δ(9)-Tetrahydrocannabinol (THC) and Its Psychoactive Metabolite, 11-OH-THC. *Drug Metab Dispos* **2019**, *47*, 249-256, doi:10.1124/dmd.118.085548.

© 2020 by the authors. Licensee MDPI, Basel, Switzerland. This article is an open access article distributed under the terms and conditions of the Creative Commons Attribution (CC BY) license (http://creativecommons.org/licenses/by/4.0/).
